# In memoriam: Professor James Gita Hakim (1954 to 2021)

**DOI:** 10.1002/jia2.25685

**Published:** 2021-03-01

**Authors:** Adeeba Kamarulzaman, Linda‐Gail Bekker, Kenneth Ngure

**Affiliations:** ^1^ Department of Medicine University of Malaya Kuala Lumpur Malaysia; ^2^ The Desmond Tutu HIV Centre Cape Town South Africa; ^3^ Departmment of Community Health Jomo Kenyatta University of Agriculture and Technology (JKUAT) Nairobi Kenya

IAS – the International AIDS Society – alongside the HIV community, mourn the untimely loss of Professor James Gita Hakim, MBChB, MMed, MMedSci, FRCP, PGDipHPE to COVID‐19‐related complications [1].

Characterized as a dignified gentleman, sage confidant, an excellent cardiologist and clinician, a consummate researcher and mentor, a brilliant academic, humanitarian and an all‐round wonderful human being, James was a scientific giant in the HIV community and instrumental in putting HIV research and science in sub‐Saharan Africa on the map, driving studies that had both regional and global relevance and impact.

Born in Sudan and trained in Uganda, Kenya, Germany and Australia, James was passionate about enhancing clinical and research capacity in his adopted country, Zimbabwe until his untimely death on 26 January 2021.

As a tireless scholar, James was a Professor of Medicine, the past Chair of Medicine at the University of Zimbabwe (UZ), where he maintained a diverse and expansive interest in HIV research including antiretroviral therapy, prevention, opportunistic infections and perinatal infection. He was also an Adjunct Professor at the University of Colorado Denver. He contributed to seminal HIV research through more than 180 publications and international communications and was honoured with the Ward Cates Spirit Award in 2019 for his outstanding commitment and leadership to health as a right, scientific excellence and generosity in mentoring the next generation of scholars.

James led multiple research programmes and networks with funding from multiple agencies, including the Medical Research Council Clinical Trials Unit (MRC CTU) (UK), the National Institutes of Health (NIH) (USA), the European and Developing Countries Clinical Trials Partnership (EDCTP) (Europe), the Rockefeller Foundation, the Department for International Development (DFID) (UK) and the Wellcome Trust. In 2001, he established the University of Zimbabwe Clinical Research Centre, which has collaborated with the MRC CTU at the University College London on many trials. He was a Principal Investigator/Site Leader within the Harare‐based UZ‐University of California San Francisco Clinical Trials Unit. Through the Development of Anti‐Retroviral Therapy (DART) study, he was instrumental in the introduction of ART in low resource settings, that has been associated with long‐term HIV control in Zimbabwe and the region [2]. He was the local Principal Investigator for the successful HPTN 052 study and involved in several Kirby Institute trials in HIV therapy for more than a decade. Most recently he was a site leader of the Coronavirus Outcomes in HIV Evaluation in Resource‐Limited Settings study (COHIVE) for the Kirby Institute [3].

James was perhaps most esteemed for his dedication and passion towards teaching, mentorship and capacity building within the African Region. He inspired many of his colleagues, holding up a mirror to the rest of us to do our part. To this end, he was the Principal Investigator (PI) of the Zimbabwe Medical Education Partnership Initiative (MEPI) award (2010 to 2016) and the Chair of the MEPI PI Council (2014 to 2015) which brought together the 13 Sub‐Saharan African MEPI medical schools [4]. He was additionally Programme Director of the Promote Excellence in Research and Faculty Enhanced Career Training (PERFECT) Programme, an NIH‐sponsored advanced junior faculty research training initiative at the University of Zimbabwe College of Health Sciences (UZCHS). His humble and patient approach to mentorship of junior investigators was a gift to those he taught and truly reflective of his calm and thoughtful nature.

James served as an elected African representative on the IAS Governing Council from 2016 to 2020. He saw the value of leveraging conferences in the region as a means of showcasing African HIV research and was instrumental in organizing the regional HIV/AIDS conferences ICASA and INTEREST and was to be a co‐Chair of ICASA 2021. He was the driving force behind bringing a regional IAS Educational Fund meeting, scientific writing workshop and press fellowship programme to Zimbabwe in June 2019.

James was an extraordinary professional, a man who was loved by patients and respected by colleagues not only in Zimbabwe but also in the region and around the world. In meetings, you had great confidence that when he spoke it would be deeply considered, rational, compassionate and certain to move the discussion forward. At the IAS, we will most deeply miss his friendship. He was a reliable and streadfast force, that could be counted on at any time. A beloved family man, anybody who knew him was touched by his modesty, wisdom, kindness and terrific sense of humour.

Forty years on from the onset of AIDS, James found himself working in a new pandemic in his adopted homeland of Zimbabwe. That he was attending to patients in COVID‐19 wards shortly up until his passing is unsurprising to us. James leaves a legacy that cannot help but inspire all of us going forward.

## Competing interests

The authors have declared no conflict of interest.

## AUTHORS’ CONTRIBUTIONS

All authors have contributed to the preparation of the manuscript, read and approved the final draft.

## Author information

Dr Adeeba Kamarulzaman, IAS President and Professor of Medicine at the Faculty of Medicine at the University of Malaya. Dr Linda‐Gail Bekker, Director of the Desmond Tutu HIV Centre at the University of Cape Town and Chief Research Officer of the Desmond Tutu Health Foundation. Past IAS President. Dr Kenneth Ngure, Associate Professor of Global Health and the Chair Department of Community Health of Jomo Kenyatta University of Agriculture and Technology (JKUAT), Kenya, Affiliate Associate Professor, Department of Global Health University of Washington, IAS Governing Council Member.
